# No evidence of firstly acquired acute hepatitis C virus infection outbreak among HIV-infected patients from Southern Spain: a multicentric retrospective study from 2000-2014

**DOI:** 10.1186/s12879-016-1826-2

**Published:** 2016-09-15

**Authors:** Karin Neukam, Pompeyo Viciana, Guillermo Ojeda-Burgos, Marcial Delgado-Fernández, María J. Ríos, Juan Macías, Dolores Merino, Antonio Collado, Francisco Téllez, Juan A. Pineda

**Affiliations:** 1 Unit of Infectious Diseases and Microbiology, Hospital Universitario de Valme, Avenida de Bellavista S/N, 41014 Seville, Spain; 2 Instituto de Biomedicina de Sevilla (IBiS), Avenida Manuel Siurot S/N, 41013 Seville, Spain; 3 Infectious Diseases, Microbiology and Preventive Medicine, Hospital Universitario Virgen del Rocío, Avenida Manuel Siurot S/N, 41013 Seville, Spain; 4 Unit of Infectious Diseases, Hospital Universitario Virgen de la Victoria, Campus Universitario Teatinos, 29010 Malaga, Spain; 5 Unit of Infectious Diseases, Hospital Regional de Málaga, Avenida de Carlos Haya, S/N, 29010 Malaga, Spain; 6 Unit of Infectious Diseases, Hospital Universitario Virgen de la Macarena, Avenida Dr. Fedriani 3, 41007 Seville, Spain; 7 Unit of Infectious Diseases, Complejo Hospitalario de Huelva, Ronda Exterior Norte S/N, 21005 Huelva, Spain; 8 Unit of Infectious Diseases, Hospital Torrecárdenas, Calle Hermandad Donantes de Sangre S/N, 04009 Almeria, Spain; 9 Unit of Infectious Diseases and Microbiology, Hospital La Línea, AGS Campo de Gibraltar, Avenida Menéndez Pelayo 103, 11300 La Linea de la Concepcion, Spain

**Keywords:** Hepatitis C, HIV, Homosexuality, Epidemics, Injecting drug use, Infectious diseases

## Abstract

**Background:**

Acute hepatitis C virus (HCV) infection (AHCVI) outbreaks have been described recently within defined areas worldwide among HIV-infected homosexual men. This study aims to describe the cumulative frequency and incidence of firstly acquired AHCVI in an HIV-infected population in Southern Spain.

**Methods:**

This is a retrospective study conducted at the Infectious Diseases Units of eight hospitals in Andalusia, Southern Spain. Primary AHC was considered as HCV immunoglobulin G antibody seroconversion. The time of infection was considered the moment between the last negative and the first positive HCV antibody determination.

**Results:**

A total of 23 cases of primary AHCVI have been detected from 2000 to 2014. Incidence rates [IR; 95 % confidence interval (CI)] were 0.036 (2.272–0.054) per 100 person-years (py) in the overall population over a follow-up period of 64170 py. Of the 22 (95.7 %) male subjects, 21 (95.5 %) had acquired AHCVI by homosexual contact, the IR (95 % CI) was 0.039 (0.024–0.06) per 100 py in this subpopulation. There was no evidence of an increase of AHCVI IR. The incidence of AHCVI was slightly lower between 2000 and 2004 as compared to 2005–2009 [IR ratio (IRR) of 8.8 (95 % CI: 1.279–378.794; *p* = 0.01)] but reached a plateau afterwards [IRR between 2010 and 2014 versus 2005–2009: 0.727 (0.286–1.848; *p* = 0.5)]. The median (Q1-Q3) time between the last negative anti-HCV and the first positive anti-HCV determination was 4.7 (1.9–11.2) months. Peak (Q1-Q3) ALT and total bilirubin values during AHCVI were 496 (291–656) IU/mL and 1.15 (0.9–1.98) mg/dL, respectively.

**Conclusions:**

In contrast to what has been reported from other areas, the incidence of primary AHCVI in the HIV-infected population is stable in Southern Spain and there is no evidence of an epidemic, in spite of the high prevalence of HIV/HCV-coinfection in this area.

## Background

In the last decade, various outbreaks of acute hepatitis C virus infection (AHCVI) have been described among HIV-infected individuals worldwide [1–10]. These epidemics have affected mainly men who have sex with men (MSM) and have led to an increase in the incidence of hepatitis C virus (HCV) infection in some particular areas [5, 6]. Additionally, recent studies conducted in very specific areas within large Spanish cities have reported an increase in the number of cases of AHCVI among HIV-infected MSM [7, 8].

To date, no cases of AHCVI among the HIV-infected population from Southern Spain have been published, in spite of the fact that in this area over one third of HIV-infected subjects currently bear an active HCV coinfection [11]. This might suggest that, whatever the reason, the occurrence of acute HCV infection outbreaks among HIV-infected MSM is subject to regional variations. However, cases of acute HCV infection may also go unnoticed, since many of them are subclinical. Because of this, underreporting of AHCVI could have underlied the lack of detection of outbreaks of this disorder in our area. It is to note that approximately 40 % of the HIV-coinfected population in the Southern Spanish region of Andalusia does not show positive serology for HCV and is thus susceptible for AHCVI. To provide insight on this point, this study aimed to determine the number of cases of firstly acquired AHCVI infection among HIV-infected patients seen at eight Southern Spanish hospitals, who attend a vast population of HIV-infected individuals with high prevalence of HCV coinfection.

## Methods

### Study population

This retrospective study was conducted in the Infectious Diseases Units of eight hospitals throughout the Southern Spanish provinces of Almeria, Cadiz, Huelva, Malaga and Seville. All HIV-positive patients seen from 2000 to 2014 and who showed negative plasma IgG antibody against HCV (anti-HCV) were included. In our area, all HIV-infected patients are cared for at public hospitals, where they attend at least once every 6 months, and may ask for additional visits when required or at their own decision. In all this institutions, routine blood parameters, including liver function test are conducted every visit. Also, HCV serum antibodies are determined at the first visit and every 12–18 months, as well as when clinically appropriated, in previously seronegative patients. All cases of firstly acquired AHCVI in HIV-infected patients diagnosed in the above-stated institutions according to the below-stated criteria were analyzed retrospectively.

### Definition of firstly acquired *AHCVI*

An episode of AHCVI was diagnosed in patients with confirmed prior absence of anti-HCV if i) Anti-HCV seroconversion or a 10-fold increase in ALT levels above the upper limit of normality (ULN) along with detectable plasma HCV-RNA were observed; ii) Acute hepatitis B virus and hepatitis A virus infection were ruled out by negative IgM anti-HBc and IgM anti-HAV tests and iii) Toxic acute hepatitis was reasonably excluded. The date of AHCVI was estimated as the midpoint between the date of the last anti-HCV negative result and the date of the first positive determination or when HCV-RNA was detected for the first time.

### Laboratory determinations

Serological determinations were conducted by enzyme immunoassay (ADVIA Centaur XP, Siemens Healthcare Diagnostics S.L. Tarrytown, NY, USA or ELECSYS® Anti-HCV II, Roche Diagnostics, Basel, Suisse). Plasma HCV RNA was quantified as described elsewhere [12]. HCV genotype was determined using the VERSANT HCV Genotype 2.0 kit (Siemens Healthcare, Erlangen, Germany).

### Statistical analysis

Descriptive statistics of demographic factors, HIV transmission route and analytical values were carried out. Incidence rates (IR) and the 95 % confidence intervals (CI) of firstly acquired AHCVI were calculated and presented as cases per 100 person-years (py). Study periods were defined as followed: 2000–2004 (period 1), 2005–2009 (period 2) and 2010–2014 (period 3). Subsequently, based on Poisson regression, IR ratios (IRR) were determined in order to evaluate the relative risk for acquisition of AHCVI between the different study periods, as well as for possible geographic differences were calculated. Analyses were carried out by means of the SPSS statistical software package release 23.0 (IBM Corporation, Somers, NY, USA) and STATA 9.0 (StataCorp LP, College Station, TX, USA).

### Ethical aspects

The study was designed and performed according to the Helsinki declaration and was approved by the Ethics Committee of the Valme University Hospital (Seville, Spain). All patients gave their written informed consent.

## Results

A total of 23 cases of primary AHCVI were detected. All of them were observed in five of the eight participating hospitals: 10 cases were reported from Malaga and the remaining 13 individuals were identified in Seville. The characteristics of the patients at the moment of AHCVI diagnosis are listed in Table 1.

**Table 1:** Characteristics of the patients at the moment of diagnosis of acute HCV infection

Characteristic	Value
Age (years)^a^	43.2 (34–46.1)
Male gender, n (%)	22 (95.7)
Race, n (%)	
Caucasian	23 (100)
Risk factor for HCV acquisition, n (%)	
Heterosexual contact	1 (4.3)
MSM	21 (91.3)
PWID	1 (4.3)
HCV genotype, n (%)	
1 (1a/1b)^b^	19 (82.6) [10 (43,5)/ 2 (8,7)]
3	2 (8.7)
4	2 (8.7)
HCV RNA (log10 IU/mL)^a^	6.08 (5.37–6.98)
ALT (IU/mL)^a^	367 (234–614)
AST (IU/mL)^a^	181 (93–646)
Total bilirubin (mg/dL)^a^	1.1 (0.72–3.39)

*MSM* men who have sex with men, *PWID* people who inject drugs

^a^Median (interquartile range); ^b^7 subject did not have HCV genotype 1 subtype determination

**Fig. 1:**
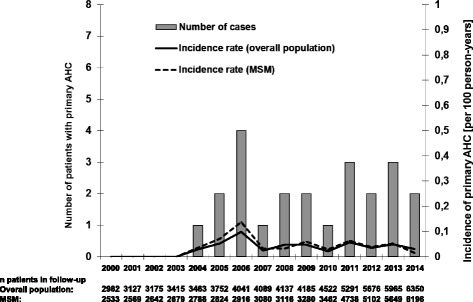
Number and incidence of patients rate with diagnosis of primary acute hepatitis C virus infection (AHCVI) among HIV-infected individuals according to the years of infection. MSM: men who have sex with men

**Fig. 2:**
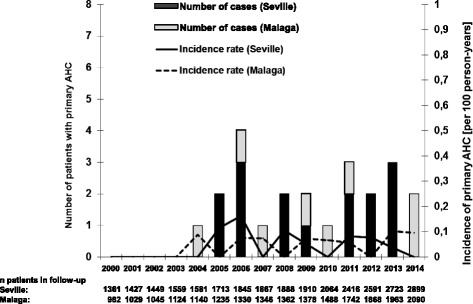
Number and incidence of patients with diagnosis of primary acute hepatitis C virus infection (AHCVI) among HIV-infected individuals according to the city where diagnoses took place

No AHCVI episode was observed before 2004. The majority (91.3 %) of the patients were MSM, none of whom had any history of injecting drug use during follow-up. The distribution of patients according to the years of seroconversion is depicted in Fig. 1. Over a follow-up period of 64170 py from 2000 to 2014, the overall IR (95 % CI) was 0.036 (2.272–0.054) per 100 py. The IR (95 % CI) for second and the first study period were 0.054 (0.027–0.097) per 100 py versus 0.006 (0.001–0.034) per 100 py, resulting in an IRR of 8.8 (95 % CI: 1.279–378.794; *p* = 0.01). The IRR between the third (IR 0.039 per 100 py; 95 % CI: 0.02–0.071) versus the second period was 0.727 (0.286–1.848; *p* = 0.5). In the subpopulation of MSM, the overall IR (95 %CI) was 0.039 (0.024–0.06) per 100 py. The IR (95 % CI) for the first, second and third study periods were 0.008 (0.001–0.041) per 100 py, 0.066 (0.032–0.121) per 100 py and 0.04 (0.19–0.073) per 100 py, respectively. The corresponding IRR were 8.682 (95 % CI: 1.235–376.792; *p* = 0.012) for the second versus the first period and 0.605 (95 % CI: 0.226–1.62; *p* = 0.27) for the third versus the second period. The IR for each study year are shown in Fig. 1. A subanalysis to compare the IR between Malaga and Seville revealed IR of 0.048 and 0.044, respectively (IRR: 0.937; 95 % CI: 0.38–2.388, *p* = 0.872). Figure 2 shows the number and incidence of primary AHCVI per year.

The median (interquartile range, IQR) time between the last negative anti-HCV and the first positive anti-HCV determination was 4.7 (1.9–11.2) months. The median (IQR) peak ALT values and total bilirubin levels during AHCVI were 496 (291–656) IU/mL and 1.15 (0.9–1.98) mg/dL, respectively. Fifteen (65.2 %) patients showed 10-fold or greater increases in ALT levels above the ULN throughout the episode of AHCVI. Likewise, 6 (26.1 %) individuals showed total bilirubin levels above 2 mg/dL at some point during acute infection.

## Discussion

In this study, the number of firstly acquired AHCVI episodes among HIV-infected patients seen at 8 hospitals from Southern Spain has not experienced a progressive increase, as observed in other areas [3, 6–8]. Conversely, the number of cases diagnosed after 2003 has remained stable. This data strongly suggest that currently there is no ongoing outbreak of AHCVI among HIV-infected patients in Southern Spain.

A recent analysis of a nationwide Spanish cohort of HIV-infected individuals suggested an augmented HCV follow-up testing of possible HCV seroconversion in the HIV-infected population in the last years in our area [13]. In spite of this, the incidence of AHCVI observed in the present study did not change during the last years. It is, however, to note that no cases of AHCVI were reported between 2000 and 2003, which resulted in a slightly lower IR for the first study period. Still, the increase in the number of cases from before to after 2003 was minimum, with a peak of 4 patients per year. Furthermore and in contrary to what was observed in other cities, [1–10] the incidence of primary AHCVI has been stable throughout the last decade, with no evidence of a trend towards an increase.

Considerable increases of the number AHCVI episodes have been reported from very specific areas of Spain. In this context, a retrospective study conducted from 2008 to 2012 in an outpatient clinic of Madrid reported 19 cases of AHCVI, seven of which were diagnosed in 2012 [7]. Likewise, a case report derived from a clinic attending the largest MSM community in Madrid, reported four cases of recently acquired AHCVI among MSM HIV-infected patients [9]. Additionally, a retrospective analysis conducted in HIV-infected individuals seen from 2003 to 2015 in a clinic from Barcelona, which also attend a large community of MSM, reported an important increase of AHCVI episodes. Thus, while only 3 patients were diagnosed for AHCVI between 2003 and 2006, approximately one episode per month was registered from 2007 to 2011, reaching a peak of almost 30 diagnoses in 2012 [8]. Clearly, this tendency is not observed in our study, despite the presence of a MSM community especially in the costal area of Southern Spain and in the Seville area [11]. Interestingly, there was no also no difference in the incidences of AHCVI when comparing the inland city of Seville and the coastal city of Malaga.

The lack of an epidemic of AHCVI in an area where, on the one hand, approximately 60 % of the HIV-infected patients are positive for HCV antibodies [11] and, on the other hand, only 20 % show active HCV-infection, [14] is an intriguing point. This fact may have different reasons. On the one hand, it has been speculated that host genetic factors play a role in the susceptibility of HCV infection [15]. However, the role of host genetics in this context remains unclear and it is unlikely that there is a significant genetic diversity between the populations of Central/Northern Spain and the herein analyzed individuals [16]. On the other hand, the results of phylogenetic analyses of the viral strains and a disproportionately high frequency of HCV genotype 4 infection suggest a clustering of strains causing AHCVI outbreaks in HIV-infected MSM [2, 4, 7, 17, 18]. Likewise, in HIV-infected MSM with AHCVI in Spain, the proportion of acute HCV genotype 4 infections is higher than expected when compared to the prevalence of HCV genotype 4 in chronically infected populations [7, 8]. Conversely, in the present study acute infection with genotype 1 was predominantly observed, standing in accordance with the HCV genotype distribution commonly observed among HIV/HCV-coinfected individuals seen in our area [11]. Thus, this observation adds up to the conclusion that the situation in Southern Spain is different to what is observed elsewhere. Finally, differences in sexual risk behaviour may account for the lack of an epidemic in our setting. While transmission of HCV is rare in HIV-positive population, [6] and even more uncommon in HIV-negative heterosexual couples, certain practices often carried out by MSM, such as unprotected anal sex with bleeding, fisting or group sex have been associated with HCV transmission [3, 19, 20]. Additionally, in MSM, HIV-coinfection itself might favour sexual HCV transmission [21]. Although no comparative studies on sexual risk behaviour have been conducted between different areas, data obtained from HIV-infected MSM in our setting suggest that high risk behaviour is also common among them [22]. In our opinion, certain HCV strains particularly infective through transmucosal way may spread among a specific MSM subset with high risk practises leading to an outbreak of AHCVI. It is unlikely that this chain of events has taken place in our area so far. This is supported by another Southern Spanish study conducted in HIV-infected patients who had achieved treatment-induced HCV clearance and which found a very low incidence rate of reinfection with HCV [23]. However, this point requires further investigation but, if confirmed, it would have important implications for preventing HCV transmission among HIV-infected MSM.

The main limitation of this study is its retrospective design. Indeed, since episodes of AHCVI are frequently asymptomatic, [24] as in was the case in our patients, we cannot rule out that some cases could have gone unnoticed. However, it is unlikely that diagnosis of AHCVI was missed to a great extent in our hospitals in the last decade. In fact, HCV serology is usually performed at the first visit and sequentially afterwards in order to diagnose HCV infection at the Infectious Diseases Units in our area. Still, a prospective study to evaluate the incidence of AHCVI in HIV coinfection is warranted. This study should include a detailed questionnaire about sexual practices that enabled to compare data with those already carried out in other regions.

## Conclusions

In conclusion, the number of cases of primary AHCVI in HIV-infected patients in Southern Spain is both low and stable. Prospective studies to determine the incidence of AHCVI, as well as the reasons for the lack of an epidemic outbreak in this setting, are necessary. These data are crucial for preventing AHCVI. In the meantime, awareness and sequential screening for HCV antibodies in seronegative patients are needed.

## Abbreviations

AHCVI: Acute hepatitis C virus infection
anti-HCV: Plasma IgG antibody against hepatitis C virus
HCV: Hepatitis C virus
IR: Incidence rate
IRR: Incidence rate ratio
MSM: Men who have sex with men
ULN: Upper limit of normality
